# Atomistic molecular dynamics simulations of tubulin heterodimers explain the motion of a microtubule

**DOI:** 10.1007/s00249-021-01553-1

**Published:** 2021-07-02

**Authors:** Alexandr Nasedkin, Inna Ermilova, Jan Swenson

**Affiliations:** Department of Physics, Chalmers University of Technology, SE 41296 Göteborg, Sweden

**Keywords:** Microtubules, Molecular dynamics, Diabetes, Protofilament, Equilibrium dynamics, Small drugs, Alzheimer’s disease

## Abstract

Microtubules are essential parts of the cytoskeleton that are built by polymerization of tubulin heterodimers into a hollow tube. Regardless that their structures and functions have been comprehensively investigated in a modern soft matter, it is unclear how properties of tubulin heterodimer influence and promote the self-assembly. A detailed knowledge of such structural mechanisms would be helpful in drug design against neurodegenerative diseases, cancer, diabetes etc. In this work atomistic molecular dynamics simulations were used to investigate the fundamental dynamics of tubulin heterodimers in a sheet and a short microtubule utilizing well-equilibrated structures. The breathing motions of the tubulin heterodimers during assembly show that the movement at the lateral interface between heterodimers (wobbling) dominates in the lattice. The simulations of the protofilament curvature agrees well with recently published experimental data, showing curved protofilaments at polymerization of the microtubule plus end. The tubulin heterodimers exposed at the microtubule minus end were less curved and displayed altered interactions at the site of sheet closure around the outmost heterodimers, which may slow heterodimer binding and polymerization, providing a potential explanation for the limited dynamics observed at the minus end.

**Supplementary Information:**

The online version contains supplementary material available at 10.1007/s00249-021-01553-1.

## Introduction

Biological cells and their components have always been of high interest in the soft matter community (Lam et al. 2014; Edozie et al. 2019; Pan et al. 2014; Gallová et al. 2004; Murtola et al. 2009). Various model systems have been created by scientists to understand vital processes in cells. Bio-membranes, proteins, peptides, ionic channels and nucleic acids are very popular to study by both experimental and simulation methods (Ayton et al. 2007; Murugova et al. 2020; Nakabeppu et al. 2007; Subczynski and Pasenkiewicz-Gierula 2020; Kuhrova et al. 2019; Ermilova et al. 2017; Nasedkin et al. 2017). Researchers have tried to relate various diseases to certain behaviors of proteins (Aguzzi and O’Connor 2010; Abriel et al. 2013), to compositions of lipid bilayers (Ntarakas et al. 2019; Ermilova 2019), to structures and behavior of nucleic acids (Maharana et al. 2018; Roth et al. 2011) etc. Nevertheless, cures for many diseases are still not found, which raises the importance of investigating other cell compounds such as microtubules.

**Fig. 1:**
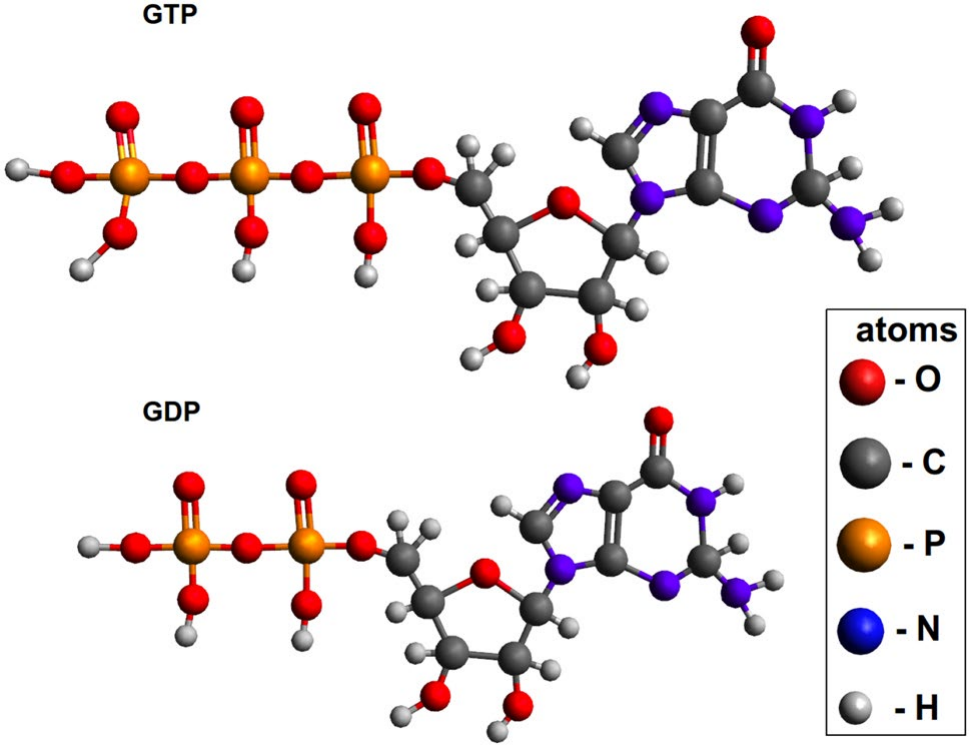
Guanosine-5’-triphosphate (GTP) and guanosine-5’-diphosphate (GDP)

Microtubules consist of tubulin heterodimers and they are an essential part of the cytoskeleton (Goddard et al. 1994; Kapitein and Hoogenraad 2015; Fojo 2009). Structurally, they are hollow cylinders with walls that are formed by laterally bound parallel filaments of tubulin, called protofilaments (PF) (Jibu et al. 1994). Microtubules and their dynamics play a big role in neurodegenerative diseases (Matamoros and Baas 2016). Particularly, they are required for the construction of axons and dendrites during the life of a neuron. In neurodegenerative diseases the mass of the microtubule is reduced, its electrostatic and a microtubule-mediated transport get disintegrated (Matamoros and Baas 2016). Such a behavior could be either the consequence or the cause of a disease.

Microtubules are known to be involved in the regulation of localization and availability of insulin in pancreatic $\beta$-cells, which makes microtubules interesting candidates for the drug design against diabetes (Bracey et al. 2020). Moreover, these species are often seen associated with tumor suppressors in breast cancer and therefore can become biomarkers for this disease (Rodrigues-Ferreira et al. 2020; Rong et al. 2004).

**Fig. 2:**
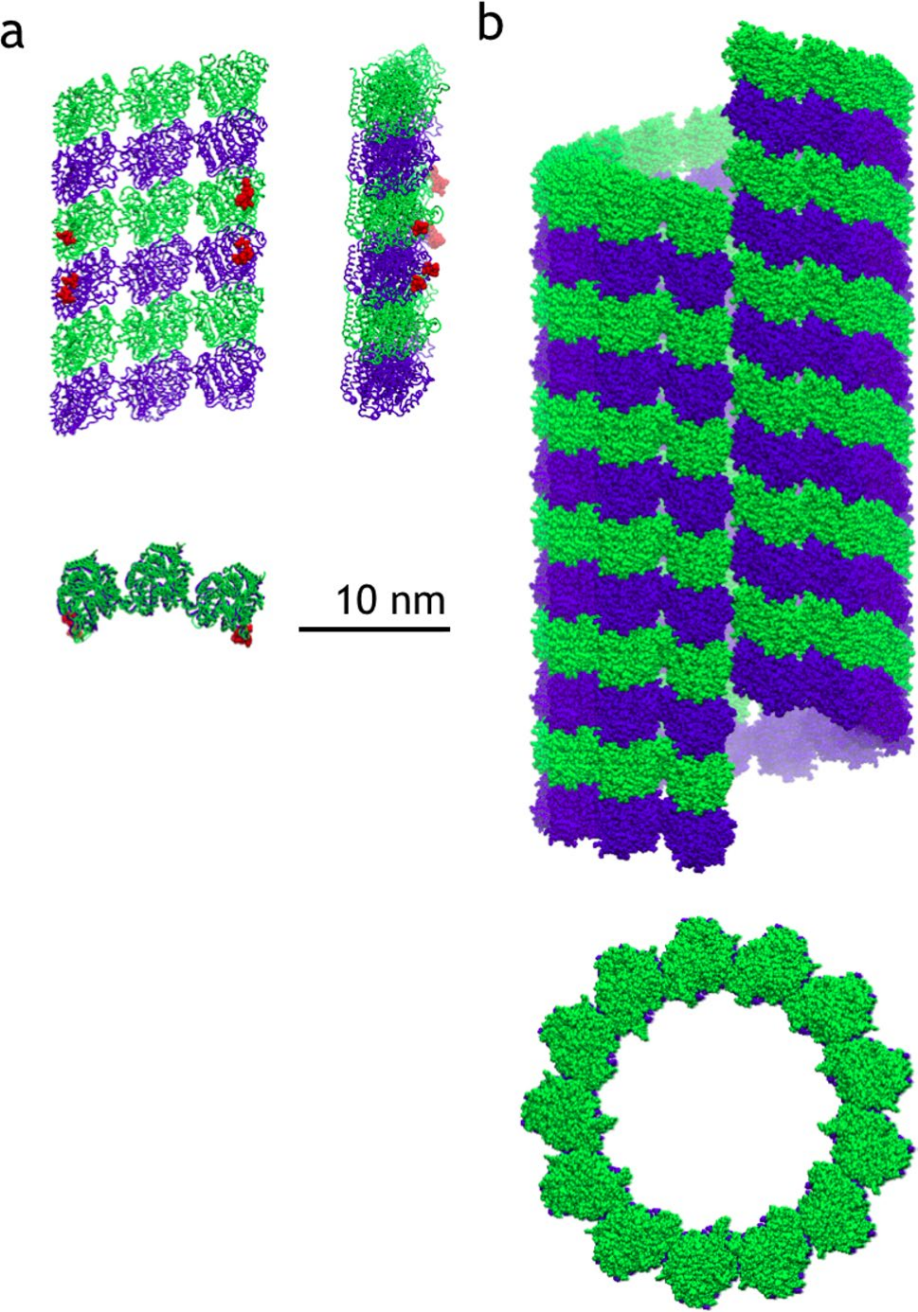
Systems simulated in this work to scale. **a** A protofilament (PF) sheet of nine heterodimers as viewed from three sides. Red ball-and stick representation shows residues constrained in their movements during simulations. **b** A microtubule consisting of 78 heterodimers (12 monomeric layers) as viewed from the side and from the plus-end

Early studies by cryo-electron microscopy revealed a significant diversity in the structure of growing and shrinking microtubule tips, as well as the structures of the positive and negative ends (Simon and Salmon 1990; Mandelkow et al. 1991; Chretien et al. 1995). Subsequently revealed structures of the microtubule structural unit, $\alpha \beta$-tubulin heterodimer (Nogales et al. 1998), shed light on the large variation of forms adopted by the polymerized microtubule tip, as well as on other shapes of polymerized tubulin. The heterodimer is nearly straight in the microtubule lattice, yet it can bend at the interface between constituting monomers. Thus, microtubular dynamics is highly dependent on the conformational changes on a level of a single tubulin heterodimer. In this perspective, explorations of the conformational space sampled by tubulin may facilitate the construction of more integrative models for tubulin assemblies.

One of the best ways to sample and investigate conformational changes of tubulin heterodimers is to study them using molecular dynamics (MD) simulations. Sampling in MD simulations can be set to e.g. picosecond time range. Such a time-scale of motions is of a high interest for the kinetics in drug design. Large-scale collective motions that are responsible for protein functions, such as substrate binding and reaction-associated conformational changes, can also be investigated employing MD simulations. The concept of essential dynamics (ED) was originally proposed in the early history of MD simulations for extracting such motions from the results of simulations (Amadei et al. 1993). In its essence, the ED postulates that the eigenvector hyperspace of protein motions could formally be split into an eigenvector subspace essential for protein functions and to a subspace of constrained harmonic motions existing merely as a noise. The sum of eigenvalues of the essential space should be substantial in this way, showing that much of the dynamics is locked in the large-scale motions.

**Fig. 3:**
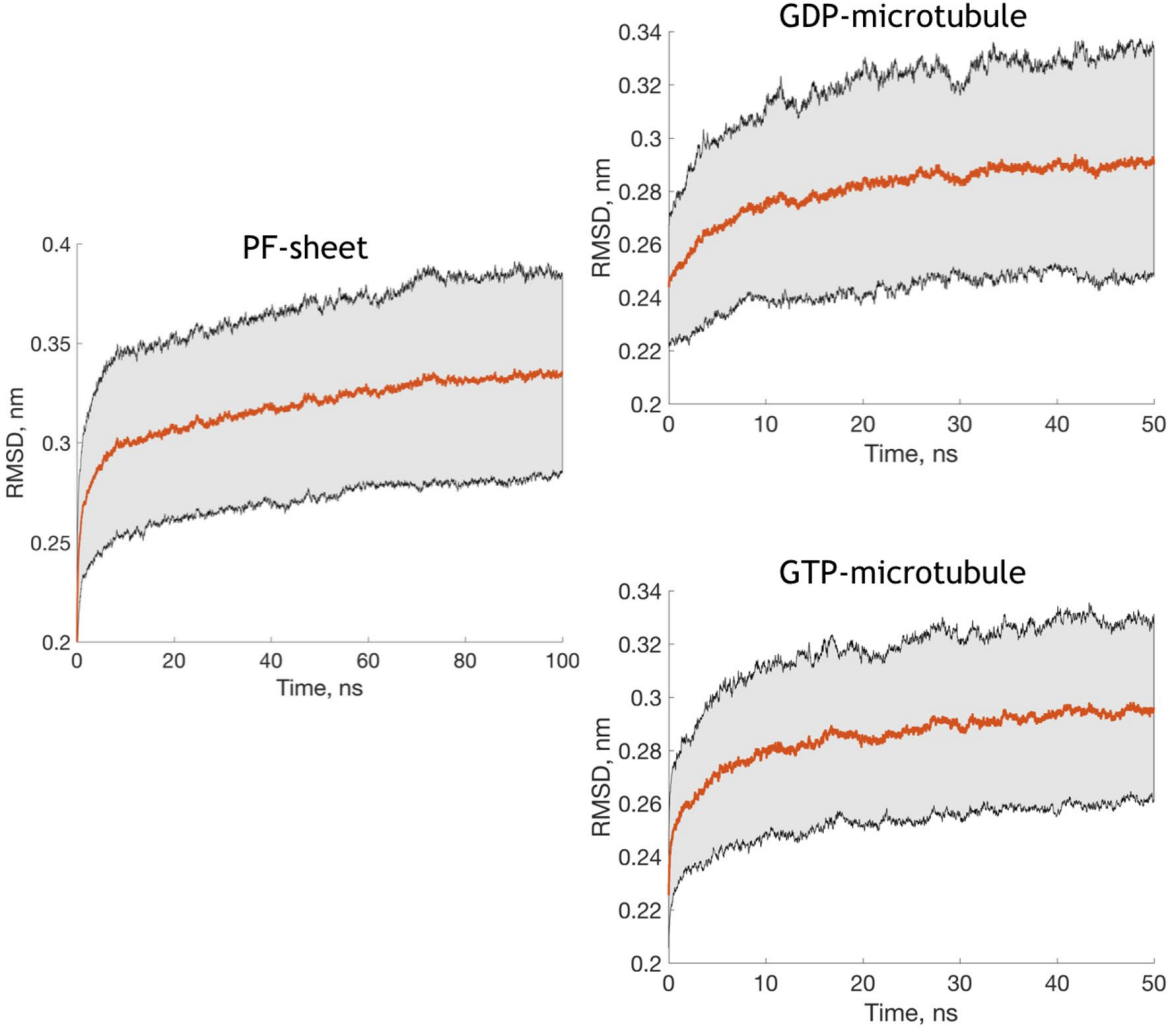
Average root mean square deviation (RMSD) of the heterodimer in simulated systems for C$_{\alpha }$ atoms. Top —PF sheet, middle—GDP microtubule, bottom—GTP microtubule. The shaded area depicts standard deviation of RMSD values

ED of tubulin has been sampled by coarse-grained anisotropic network model simulations of a microtubule (Keskin et al. 2002) and all-atom MD simulations of individual $\alpha \beta$-tubulin heterodimers (Majumdar and Ghosh Dastidar 2017). These studies identify similar principle motions, however their contributions to the total dynamics differ for different systems. This could be accounted by e.g. the difference in the computational models employed. However, investigations of the dynamics of tubulin in biological assemblies by MD simulations are essential since they will improve our understanding of dynamical changes happening upon binding to the microtubule and the possible implication of these changes regarding the interactions with other molecules.

**Fig. 4:**
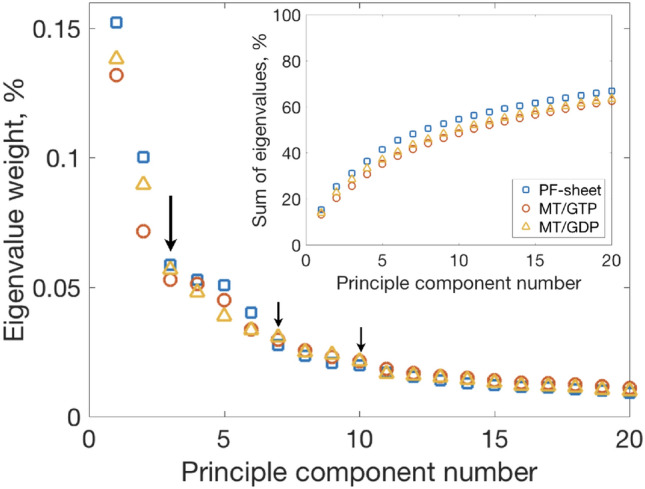
Scree plot of the first 20 principal modes. Large arrow ($\downarrow$) indicates the first kink in the scree plot and small arrows ($\downarrow$) point to the second and the third kink in the plot. The inset shows accumulated dynamics as a ratio between a sum of all modes up to the current mode and a sum of all modes

The dynamics of the entire microtubule was sampled by MD simulations several years ago in a work by Wells and Aksimentiev (2010). They built atomistic models of a small and an infinite microtubules from cryo-electron microscopy maps and investigated their dynamics under the influence of external forces.

Other studies have concentrated on a single $\alpha \beta$-tubulin heterodimer or smaller PF constructs (Majumdar and Ghosh Dastidar 2017; Theisen et al. 2013; Grafmuller and Voth 2011). The idea to reduce the dynamics of the whole microtubule to the dynamics of the $\alpha \beta$-tubulin heterodimer has been implemented to explore mechanical properties, energetics and aging of the microtubule, with impressive results obtained by Deriu et al. (2010) and Zakharov et al. (2015). For instance, Deriu et al. developed a highly coarse-grained model of the entire microtubule and carried out MD simulations with it, where the length of the microtubule was 350 nm. In their work they were able to reproduce mechanisms of its motion with a good accuracy (Deriu et al. 2010). Zakharov et al. (2015) have used advanced coarse-grained MD simulations for modeling of tubulin-tubulin interactions. They discovered that microtubule aging happens without any observable changes in the microtubule wall or tip (Zakharov et al. 2015). Yet, some differences in the behaviour of individual heterodimers in the bio-assembly may arise, which should be properly accounted by multiscale modeling (Barsegov et al. 2017).

**Fig. 5:**
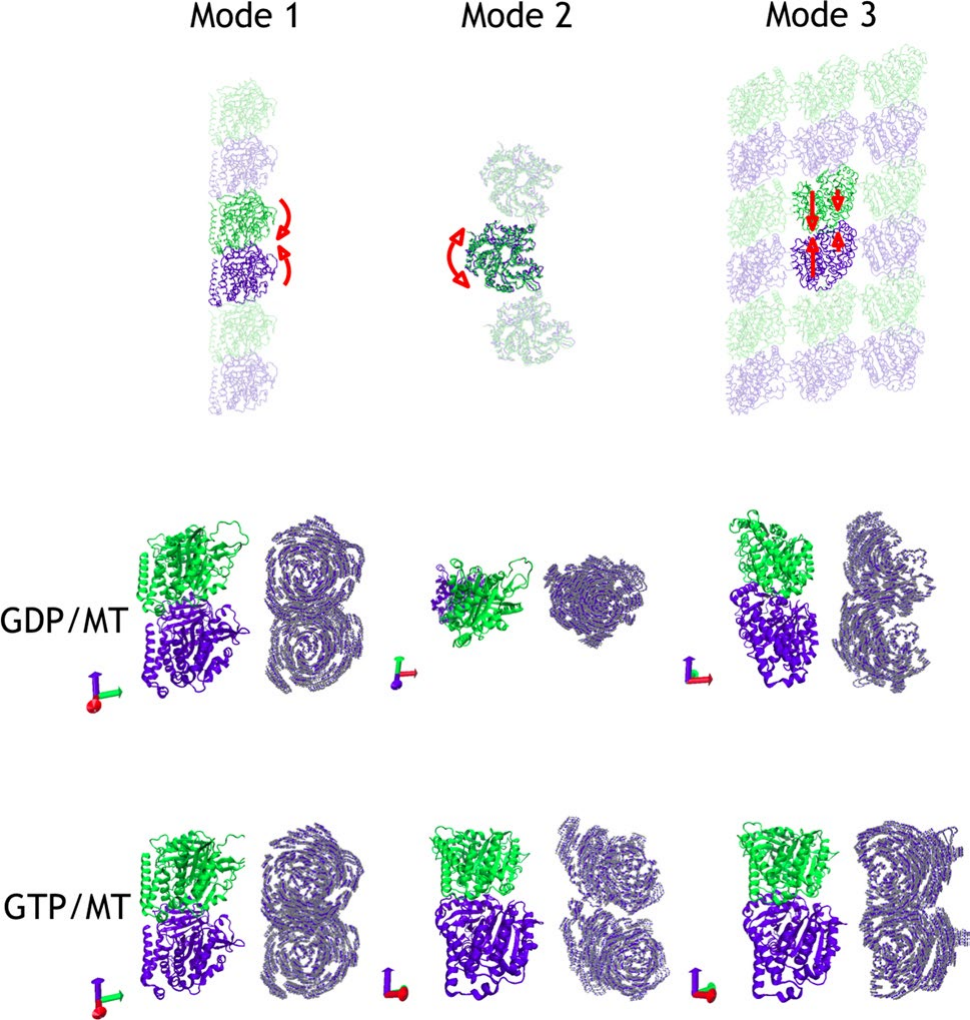
The top row shows the first three modes of $\alpha \beta$-tubulin heterodimers as seen in the microtubule lattice. Modes derived in MD-simulations from left to right: wobbling toward the microtubule axis, rotation around common monomer axis and compression along the common axis of monomers. Arrows in the compression mode qualitatively indicate the amplitude of compression for various parts of tubulin heterodimer. The middle and the bottom rows show motions of the tubulin heterodimer for the GDP and GTP-bound microtubules respectively. Two images for every mode show the tubulin heterodimer and motions as to see the largest displacement

In light of previous observations it would be valuable to follow the principle modes of the tubulin heterodimer in the PF sheet and in the microtubule by all-atom molecular dynamics simulations. In this work we try to understand the conformational space accessible to the tubulin protein and compare it to the data from other methods. As a complement to the work of Wells and Aksimentiev (2010), we use here a longer model of a pharmaceutically relevant complete microtubule with 12 monomeric layers. Instead of investigating the effects of external stress on the system, the equilibrium dynamics is studied in the current work. Comparison with dynamics of the unpolymerized heterodimer will highlight the relevance and limitations of the results obtained on unpolymerized heterodimer toward dynamics in the microtubule. Although the principle modes of axial rotation, compression and wobbling are similar when compared to dynamics of unpolymerized tubulin, we see dramatic shift in the level of the modes in the overall dynamics, such as a notable dampening of axial rotation. We discuss how this effect may play a role in how guanosine-5’-triphosphate (GTP) (see Fig.  1) binding influences polymerization of the microtubule. Furthermore, we compare the properties of tubulin dimers bound at the plus and minus ends of the microtubule and show that those exposed at the microtubule minus end were less curved and displayed altered interactions at the site of sheet closure around the outmost dimer. We suggest that these altered properties can contribute to the enhanced stability of the minus end. These data underscore the usefulness of all-atom MD simulations as a way to better understand the mechanism behind microtubule dynamic instability and, in turn, how these properties could be altered not only by microtuble binding proteins in vivo, but also by microtubule-targeting drugs of clinical relevance.

## Methods

To study large-scale conformational motions we have performed analysis of three macromolecular assemblies of tubulin, namely protofilament sheet of size 3$\times$3 heterodimers (10 trajectories, 100 ns each) and two fragments of a 13-PFs microtubules consisting of 78 heterodimers in six full turns (a microtubule circumference is $n_\mathrm{turn}$=13 monomers). In all simulated systems high concentrations of Mg$^{2+}$ and Cl$^{-}$ ions are utilized in order to emulate the environment, which is a common procedure in in vitro experiments for stabilizing microtubules (Lee et al. 1978). Furthermore, the divalent ion Mg$^{2+}$ was chosen, since it is known not to be able to penetrate through the microtubule walls due to a high free energy barrier (Shen and Guo 2018). All simulations were performed at temperature of 300 K. Electrostatic and van der Waals interactions were imposed by using cut-off scheme Verlet (Grubmüller et al. 1991) with a cut-off radius equal to 1 nm. The short-range electrostatic interactions were treated explicitly, whereas the particle-mesh Ewald scheme (Darden et al. 1993) with the fourth order (cubic) interpolation was applied to treat the long-range electrostatic interactions. Constants for temperature and pressure coupling algorithms were set to 0.5 and 2 ps respectively. The isothermal compressibility of water was set to 4.5$\times$10$^{-5}$ bar$^{-1}$.

### Molecular dynamics simulations of protofilament sheet

A macromolecular assembly of nine $\alpha \beta$-tubulin heterodimers was chosen for these simulations, see Fig.  2a. The reference structure was defined by means of cryoelectron microscopy (Alushin et al. 2014) and has PDB ID: 3J6F. Several missing residues were reconstructed by MODELLER (Šali and Blundell 1993). Specifically one residue in every terminus and residues 39–48 in the $\alpha$-tubulin were added into the atomic structure according to information in the associated sequence file. MD simulations were performed using GROMACS 2016.3 package (Abraham et al. 2015). The GROMOS 54a7 force field was chosen for the simulations (Schmid et al. 2011). Molecular topologies for molecules of GTP and guanosine-5’-diphosphate (GDP) (see Fig.  1) were taken from the Automated Topology Builder (ATB) database (Koziara et al. 2014).

The system was centered in a rectangular box of size 20$\times$10$\times$32 nm. The elongation along one axis of the protofilament sheet increases the risk of producing artifacts due to self-interactions of a biological assembly through periodic boundary conditions. To prevent such self-interactions we applied position constraints (in *x*/*y* direction) on PF interacting loops of two heterodimers, as shown in Fig.  2a. These constraints prevented rotation of the PF sheet, whereas it was able to move along the *z*-axis. Roto-translational constrains are routinely applied in PF simulations (Grafmuller and Voth 2011; Theisen et al. 2013) since such constraints should not disturb the overall dynamics of the whole unit.

The SPC water model (Berendsen et al. 1981) was utilized when the system was submerged into a water bath. Cl$^-$ and Mg$^{2+}$ ions were added to emulate the standard buffer conditions (ca. 2 mM MgCl$_2$) and to neutralize the excessive negative charge of the tubulin molecules. The resulting system contained 135 Cl$^-$, 225 Mg$^{2+}$, 176,761 water molecules and nine units for every GTP, GDP, $\alpha$-tubulin and $\beta$-tubulin molecule—in total 609,870 atoms in the system.

The system was equilibrated in several rounds before the sampling simulations. At first, energy minimization was performed to remove bad solvent contacts. Further, three dynamic NPT runs of 100 ps, 100 ps and 1 ns in run-time were performed. The integration time step for these runs was 0.5 fs, 1 fs and 1 fs, respectively. The Berendsen thermo-/barostats were used (Berendsen et al. 1984). A number of position constraints (’freeze groups’ in GROMACS terminology) were applied in these simulations. Molecules of GTP and GDP were position-constrained for all the three simulations. Protein sidechains were allowed to move in the last two simulations, particularly position constrained groups in GROMACS terminology were ’Protein’, ’Mainchain+Cb’ and ’Mainchain’ respectively for these three dynamic runs. The last frame of the final equilibration trajectory was used for the production MD runs.

Conformational dynamics of the PF-sheet was sampled by ten independent MD-simulations, each of them was 100 ns long. NPT ensemble with 1 fs integration time step and 10 ps sampling rate was applied. The V-rescale (Bussi et al. 2007) thermostat and Parrinello–Rahman barostat (Parrinello and Rahman 1981) were applied. All molecules were fully flexible in simulations, i.e. no constraint algorithms were used. Apart the above mentioned position constraints on the loops of two heterodimers were employed.

### Molecular dynamics simulations of a microtubule

The input structure of a microtubule was constructed from the reduced structure of a microtubule obtained by cryo-electron microscopy, PDB ID: 5SYF (Kellogg et al. 2017). The structure 5SYF was chosen for the high resolution and its stabilization by a small anticancer drug molecule target *Taxol*, which in its turn is effectively used in chemotherapies against various types of cancer (Rowinsky and Donehower 1995).

This structure was replicated three times along the *z*-axis, resulting in six complete turns of the tubulin heterodimers (see Fig.  2b). Stabilizing taxane molecules were removed from the structure for further simulations. The input structure was prepared in a similar way as the PF structure.

**Fig. 6:**
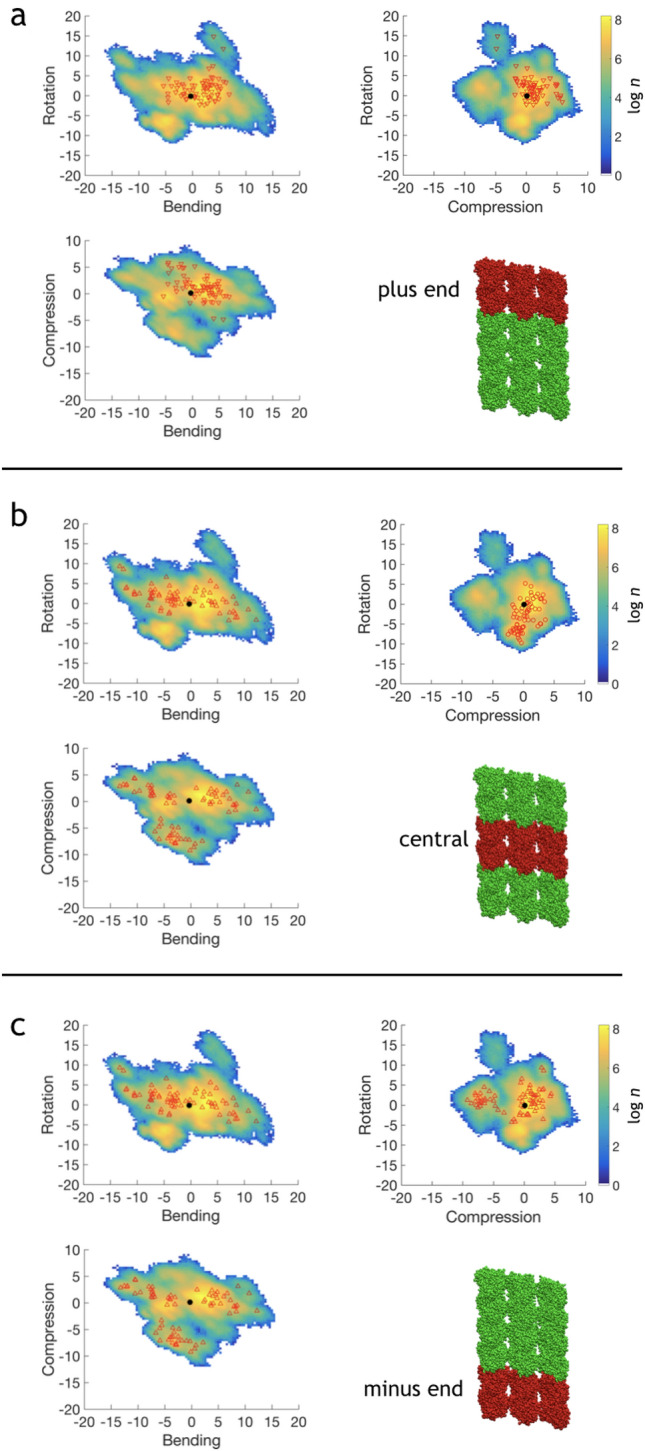
Projection of the concatenated $\alpha \beta$-tubulin heterodimer trajectory from PF-sheet simulations of the first three PC-modes. Subplots are depicting projections for various rows of heterodimers (colored in red): **a** plus-end heterodimers ($\bigtriangledown$), **b** central heterodimers ($\bigcirc$), **c** minus-end heterodimers ($\bigtriangleup$). Colorbar is given in a logarithm of the number of points in every bin of the surface. Projections are measured as an RMSD between trajectory structure and corresponding eigenvector of the PC-mode, all axes are in nm. Black solid circle ($\bullet$) is an average projection of PC-modes in the beginning of the simulation

The microtubule was centered in a rectangular box of 32$\times$32$\times$70 nm (*x*/*y*/*z*). The axis of the microtubule pointed along the *z*-direction. Unlike the PF, the microtubule was not constrained during the production run, mainly due to the much lower diffusion of the simulated microtubule fragments. After the addition of ions, the simulation box contained: 1562 Cl$^{-}$ and 2107 Mg$^{2+}$ ions, 2055,425 water molecules and 78 heterodimers for each GTP, GDP, $\alpha$- and $\beta$-tubulin, in total 6854,784 atoms.

Two systems containing a fragment of a microtubule were prepared for simulations with a difference in the nucleotide site binding, namely GDP-microtubule, where GDP is bound to $\beta$-tubulin in the nucleotide site, and GTP-microtubule, where GTP is bound to all nucleotide sites of tubulin. All the simulations started from a straight orientation of individual tubulin heterodimers.

Energy equilibration and preparatory simulations were performed similar to the PF ones. The difference was in the length of the three dynamic runs, which now were 100 ps, 1 ns and 10 ns, respectively.

The production MD simulation was 50 ns long; 1 fs time step, 10 ps sampling rate, V-rescale thermostat and Parrinello–Rahman barostat (Parrinello and Rahman 1981) were used. No constraints were applied.

### Principal component analysis

Eigenvectors, or PC-modes, were found from the principal component analysis (PCA) of the covariance matrix of the atomic coordinates by GROMACS built-in routines (*gmx covar/anaeig*). Eigenvectors are unique for a given combination of the protein and its environment, however, the derivation requires an infinite conformational sampling. Apparently, trajectories from the MD simulations often sample only a part of the conformational space. Non-ergodic MD trajectories will most likely result in ill-defined PC-modes, further questioning the reliability of the essential dynamics subset. Recently, an approach aiming to greatly improve the MD-defined PC-modes has been proposed (Cossio-Pérez et al. 2017). In this method, PC-modes are constructed from the MD-trajectory by combining several trajectories of shorter time. The concatenated trajectory contains a broader sampling of the configuration space compared to a single trajectory of an equivalent runtime, thus the ergodicity of the MD runs is improved.

Backbone atoms were used for covariance analysis. Principal modes were obtained for heterodimer structures by concatenating multiple trajectories of individual heterodimers into one lengthy trajectory. Multiple trajectories for the PF-sheet simulations were all concatenated into one, before extracting the trajectory of a heterodimer. Concatenated trajectories resulted in a 9 $\upmu$s trajectory for all MD-runs of the PF-sheet and a 3.9 $\upmu$s trajectory for the microtubule.

**Fig. 7:**
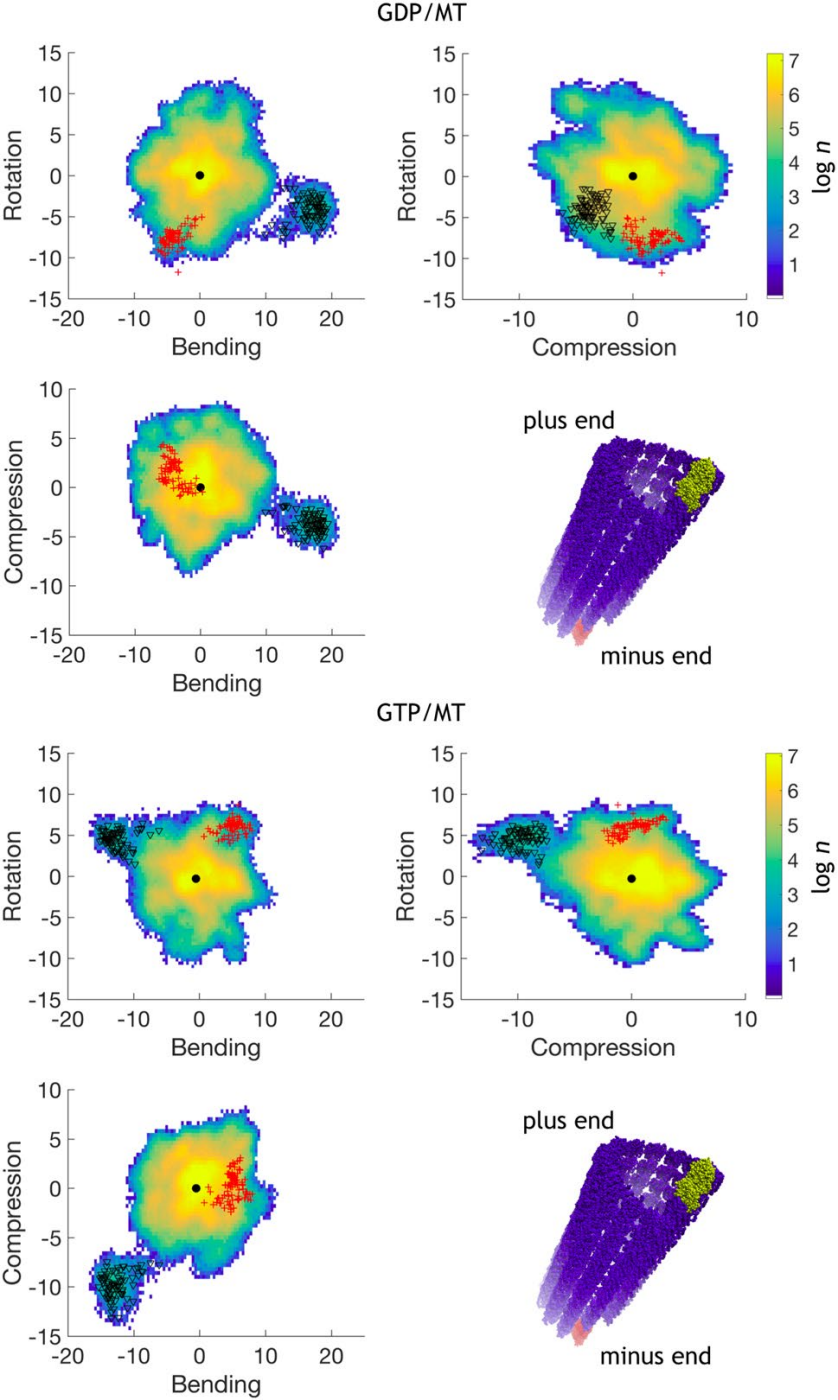
Projection of the concatenated tubulin heterodimer trajectory from microtubule simulations of GDP-bound microtubule (top panel) and GTP-bound microtubule (bottom panel) of the first three PC-modes, namely axial rotation, intradimer bending and axial compression. The map is produced by taking logarithm of population in every bin of the plot. Here “*n*” is over all considered steps in the bin. The colorbar on the right is a logarithm of the number of points in the bin of the surface. Red crosses and black triangles depict minus- and plus-end corner heterodimers respectively. The data for corner heterodimers is downsampled by block-averaging to increase visibility. Black solid circle is an average projection of PC-modes in the beginning of the simulation. All axes are in nm. Bottom right panel depicts microtubule and two corner heterodimers

**Fig. 8:**
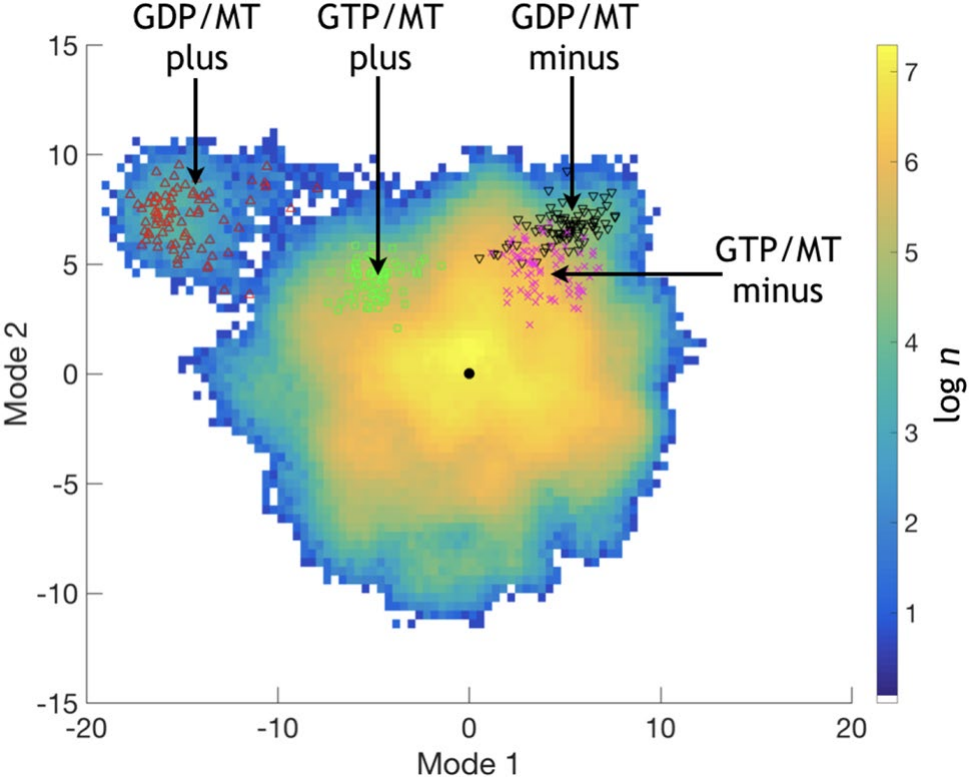
PCA on the trajectory combining GDP-MT and GTP-MT. Projections of the outmost heterodimers for every system are also shown. Here “*n*” is over all considered steps in the bin

## Results

### RMSD values

Figure  3 shows the root mean square deviation (RMSD) of heterodimers in relation to the initial structure of each simulation. The RMSD value in all three systems is slowly increasing toward larger values after a rapid increase on a short ns time-scale . A similar behavior was observed in other computational works for protofilaments and entire microtubules (Wells and Aksimentiev 2010; Deriu et al. 2010). Figure S1 in Supplementary Information shows RMSD values for dimers in GDP- and GTP-bound microtubules, computed on 3 different time intervals during the equilibration process. During 3 $\upmu$s these values fluctuate in the range of 2–4.5 Å for both microtubules. For the last 10 and 5 ns of equilibration runs RMSD values oscillate in the interval 2.8–3.8 Å. Similar fluctuations of values were observed by Hemmat et al. (2019). Such values below 5 Å during long and short time intervals indicated the stability of the MD simulations.

### Principal component analysis and scree plot

The distribution of eigenvalues by mode number, a so-called scree plot, is shown in Fig.  4 for the PF-sheet and the two microtubule systems. The eigenvalues indicate how much of the overall motions is given by each principal component mode. Qualitatively, the scree plots for both types of systems are similar with the notion that the lower number modes are more expressed in the PF-sheet system.

The scree plot is usually used to assign the number of biologically important principle modes. The common approach is to use Cattell criterion (Cattell 1966; Cattell and Vogelmann 1977), which presumes the presence of a kink in the scree plot. In this case, it is assumed that biologically relevant modes are all lower number modes before the kink. The kink itself is usually present in protein studies and more generally it appears for any high-dimensionality dataset (David 2014).

In our simulations the kink in the scree plot starts from mode number three (arrow in Fig.  4), which means there are at least three modes in the simulations that require biological assignments.

### Biologically significant PC-modes of a heterodimer

The first three modes are schematically depicted in Fig.  5. The first mode is due to wobbling toward the microtubule axis, which accounts for 15% of the motions in the PF-sheet and 12% for microtubules. The second and the third mode are similar in the PF-sheet and the GDP-microtubule (GDP-MT), where the second mode is a rotation around a common monomer axis, and the third mode is a compression along the common axis of the monomers. The second and the third mode in the GTP-MT are slightly different. The second mode in the GTP-microtubule is a rotational mode with the rotation axes of the monomers being not parallel to the common dimer axis, but tilted around 45$^{\circ }$. The third mode of the GTP-microtubule has elements of both compression and wobbling. To demonstrate the differences in the principal modes, we constructed a matrix of inner products of eigenvectors for both microtubule systems, as shown in Fig. S2 of Supplementary Information. It is visible from the figure that the first eigenvectors are very close to each other (inner product is 0.85), whereas the second and the third eigenvectors from GDP-MT are more dissimilar to their counterpart from GTP-MT (inner products are 0.64 for both). High values for non-diagonal elements is an indication of a redistribution of motions between the modes of the two MT systems.

### Projections of trajectories of the PC-modes

We have projected concatenated heterodimer trajectories of the observed PC-modes for all sets of simulations. Projections of the first three eigenvectors are shown in Figs.  6 and 7 for the PF-sheet and the microtubules, respectively. For the first three vectors those projections are relatively symmetrically shaped with their center roughly corresponding to the mean structure, i.e. zero on all axes. The maximum of the population also corresponds to the mean structure, which in turn is close to the initial structure.

PC-mode projections of plus- and minus end heterodimers noticeably differ in the PF-sheet simulations. The minus-end heterodimers are spanning larger areas in their first three eigenvectors compared to the central and plus-end heterodimers. The plus-end heterodimers are spanning a slightly larger area in the configuration space than the central heterodimers.

Projections of the first three PC-modes for the microtubule are quite featureless compared to the PF-sheet. The noticeable outlier is the projection of the outmost corner heterodimer at the plus end of the microtubule in both simulations. This heterodimer is very deformed along the bending coordinate and show relatively large deformations in rotation mode. It might be interesting to notice that the outmost dimer in GTP-MT shows a noticeable elongation which is absent in GDP-MT. Corresponding projections of the minus-end corner heterodimer do not show these types of features.

Additionally, we performed principal component analysis on the trajectory combining both GDP-MT and GTP-MT trajectories (see Fig.  8). The projection shows only one outlier corresponding to the outmost plus-end heterodimer in the GDP-microtubule. The projection for the outmost heterodimer in the GTP-MT is located half the way between the average structure and the outmost plus-end heterodimer in the GDP-MT. Projections of the outmost minus-end heterodimers are located very close to each other.

The question about the improvement of PC-modes arises after concatenating the trajectories. From Fig. S3 in Supplementary Information we could see that the value of inner product of single trajectories is around 0.15, whereas the value for 39 trajectories concatenated together is 85$\%$ or more depending on the system (GDP, GDP/GTP and GTP- bound microtubules give slightly different results, but qualitatively the trend of the plot is very similar). Thus, when we use 78 trajectories concatenated together, the precision in determination of PC-modes should definitely increase. To be on the safe side, however, we could use the value for *N* = 39 and say that our analysis gives at least 85$\%$ confidence in determination of the PC-modes.

### Bending of protofilament and microtubule

The bent orientation of heterodimers in the PF is an intrinsic property of tubulin. PF bending angle is usually defined as the angle between two lines, each of them connecting the centres of the monomers in every heterodimer (Brouhard and Rice 2014). Very large bending angles of around 20$^\circ$  are observed for example in ring complexes (Elie-Caille et al. 2007), whereas tip PFs of depolymerizing microtubule can show angles as large as 30$^\circ$  in their bending (McIntosh et al. 2008).

Bending angles were measured from frames of MD simulations using the centers of mass of each monomer as a reference point (Fig. S4 in Supplementary Information). Angles between plus- and minus oriented heterodimers with reference to the central heterodimers are shown as separate histograms in the top panel of Fig. S4 in Supplementary Information. The average bending in the PF-simulations was about 11$^\circ$, whereas in the MT-simulations the average angle was about 4$^\circ$. Similar angular distributions were observed in the computational work by Grafmuller and Voth (2011) where they investigated GDP- and GTP-bound tubulin dimers and protofilaments. Manandhar et al. (2018) simulated GDP octamers both with and without a single GTP cap layer using free energy calculations and also obtained similar distributions for intra-dimer bending angles. Such small values of bending angles imply that the microtubule is more rigid than a protofilament. This bending rigidity was also observed in other computational works (Wells and Aksimentiev 2010; Zakharov et al. 2015).

Bending angles from the PF-sheet simulations are distributed approximately equally for the minus- and plus-ends. In the microtubule, the plus-end heterodimers bend substantially more compared to the minus ends.

### Self-intermediate scattering functions for GDP- and GTP-binded microtubules

Calculation of self-intermediate scattering functions is one more way to understand the dynamics of the investigated systems. Profiles for these functions can be obtained from neutron spin-echo experiments as well as from molecular dynamics simulations (Tarek and Tobias 2000). A fast decay of the self-intermediate scattering function for a selected q-value can indicate faster motions.

A self-intermediate scattering function is a spatial Fourier transform from van Hove’s function (1954), where the latter one is determined by following Eq. ( 1):

1 $$\begin{aligned} G(r,t) = \frac{V}{N} \left\langle \rho (r_{0},t_{0}) \rho (r_{0}+r, t_{0}+t) \right\rangle . \end{aligned}$$

Here *N* is the number of particles in the system, V is its volume, $\rho$ is the number density for system, $t_{0}$ and $r_{0}$ are initial time and position of a particle, respectively.

Since simulated systems are too large for calculations of self-intermediate scattering functions only backbones and side chains of proteins were computed to characterize the motions of microtubules. Results of those calculations can be observed in Fig. S7 in Supplementary Information for the q-value equal to 1.26 Å$^{-1}$. From the presented data it can be concluded that the motion of a backbone in GDP-binded microtubule is faster than in the GTP-binded one on the time-scale above 1 ns (the curve for the GDP-binded microtubule is below the curve for the GTP-binded one). The same trend can be seen even for the side chains. Backbones and side chains are completely relaxed after about 45 ns (the self-intermediate scattering functions are equal to zero). Such a behavior on the long time scale confirms that the GTP-binded microtubule is more stable than the GDP-binded one.

## Discussion

The overall focus of this paper was to explore the principle motions of the $\alpha \beta$-tubulin heterodimer in bio-assemblies by all-atom MD simulations. Partially, the work was inspired by the notion that some drug molecules interfere with principle modes of tubulin molecules and alter the accessible conformational space of the heterodimer (Majumdar and Ghosh Dastidar 2017; Kellogg et al. 2017). Similar interference could be expected for the case of other microtubule-binding small molecules as well as proteins.

The systems simulated here were selected because of their biological interest. For example, the number of heterodimers for the microtubule nuclei has been suggested to be in the range between 5 and 20 heterodimers (Voter and Erickson 1984; Flyvbjerg and Jobs 1997; Roostalu and Surrey 2017). Thus, the simulated PF sheet could be considered a precursor of microtubule formation. The fragment of a microtubule we considered in this work has a diameter of 13 PFs, which is the number of PF most commonly seen in microtubules in vivo (Sui and Downing 2010).

The principal mode analysis we performed assign the first three modes of the tubulin as biologically significant. Projections of trajectories of these modes indicate a minimum located close to the $\alpha \beta$-tubulin heterodimer structure in the beginning of the simulation. The presence of a distinct minimum and a relatively symmetric shape of projections point to an extensive sampling of the conformational space. The center of the projections correspond to a structure in solution located close to the native crystal structure. If crystal and cryo-electron microscopy structures had been distinctly different from structures at physiological temperatures in solution, we had observed large departures from the native geometry. Moreover, one would observe an extended flare or arc in the PC-modes projections (Amadei et al. 1993). Indeed, there are large departures from the global minimum for the plus-end heterodimer located on the corners of the assembly. We associate these deformations with bending typically observed in microtubular tips (McIntosh et al. 2008) and also sampled in our simulations, as discussed below.

It is worth noticing that the PF corresponding to the outmost dimer behaves differently for GDP- and GTP bound microtubule. In the GDP-MT this PF is mostly straight and no significant differences in shape are observed compared to PFs in the body of the microtubule. In the GTP-MT system the PF extending to the outmost dimer shows bending towards the center of the microtubule. As seen in Figs. S5–S6 of Supplementary Information, the bending in GTP-MT is extending up to four dimers inside the microtubule. The nearby PFs are also affected by the bending, though to a lesser degree. This bending of the microtubule plus end could be related to the GTP-cap explaining the stability of the microtubule by the presence of GTP-bound dimers in the growing end (Howard and Hyman 2003; Galjart 2010). However, the limited length of the microtubule in our simulations prevents us from making a far-reaching conclusion about the observed capping. There is a possibility that the capping might be a result of a spontaneous bending of the microtubule. On the other hand, such dynamics is only present in the GTP-MT system and in during a relatively short time after the simulation started, which suggests that there is a GTP-related capping of the microtubule plus end.

The principle modes observed in this work qualitiatively resemble the modes derived in numerous simulation studies (Keskin et al. 2002; Gebremichael et al. 2008; Grafmuller and Voth 2011; Grafmuller et al. 2013; Bennett et al. 2009; Majumdar and Ghosh Dastidar 2017; Igaev and Grubmüller 2018; Manandhar et al. 2018), most of which identified heterodimer bending as the most dominant motion of tubulin. The wobbling mode is dominating in our simulations of all three systems, however, there are considerable differences in the character of motions for GTP- and GDP-bound microtubules (Fig. S2 in Supplementary Information).

The variation in the relative weights of the PC-modes indicates that there is some redistribution of the dynamics upon the binding into macromolecular assemblies. The PC-mode axial rotation is responsible for optimization of lateral contacts, whereas the compression mode is sensing and optimizing tubulin contacts along the length of the microtubule. Thus, for the non-polymerized tubulin much of the dynamics is locked in these two modes. Once the tubulin is bound to the assembly, lateral contacts are optimized and rigid body rotation is suppressed. In contrary, the wobbling mode is more expressed in the bound state. Movements along this mode result in bending at the interface between the connected monomers. The wobbling mode of tubulin plays a role in interactions with drugs (Mohrbach et al. 2010; Cline et al. 2012), and most probably also for the interaction with microtubule associated proteins. Furthermore, it is believed that the mode contributes to the overall microtubule bending and to the rate of catastrophe events that trigger depolymerization (Cline et al. 2012). Thus, an increase of this mode is anticipated in the polymerized form and indeed we see it in our simulations.

Bending angles extracted from the MD simulations of the PF-sheet (Figure S4a in Supplementary Information) show characteristic features for both the PF-sheet and the microtubule. The bending angle in the PF-sheet covers a vast majority of the angles observed experimentally for depolymerizing ends of the microtubule and for tubulin rings (Dima and Joshi 2008). This similarity indirectly indicates that the PF-sheet simulated here could be used as a very simplified model system for the experimentally observed flared ends of the microtubule. However, the idea to apply the results for the PF-sheet to much larger microtubular flares should be taken with caution. The distribution of bending angles for the minus end of the PF-sheet is shifted toward larger angles in the range between 10$^\circ$ and 15$^\circ$ (Fig.  6c and Fig. S4 in Supplementary Information). This is not observed experimentally for large flares (McIntosh et al. 2008). However, this bending could be specific for small tubulin constructs, such as microtubule nuclei. While the bent in the minus ends of small nuclei becomes less pronounced upon microtubule growth. Such a straightening could also result in a slowing down of the microtubule polymerization. Unfortunately, there is not much known about the structure and atomic motions in microtubule nuclei and small tubulin constructs (Roostalu and Surrey 2017; Höög et al. 2011), therefore this is highly speculative. Further, we find it notable that such a sampling of bending angles is achieved by a set of relatively short MD simulations. Thus, we hope that future studies allow for macroscopic convergence of physical quantities from microtubule MD simulations, especially given the progress in MD simulation software and algorithms (Abraham et al. 2015; Lee et al. 2016; Salomon-Ferrer et al. 2013).

Finally, we move our discussion to variations in the bending of polymerized heterodimers. Early experimental data on laser cutting and micro-needle severing of microtubules (Walker et al. 1989; Tran et al. 1997) shows that the plus-end of a microtubules rapidly depolymerizes after a cut, whereas the minus-end does not shorten. This effect is phenomenologically explained by a model of a GTP-cap formed at the plus end of the growing microtubule, such that the binding with GTP promotes straightening of the $\alpha \beta$-tubulin heterodimer (Melki et al. 1989). Later crystallographic data for GTP-bound heterodimers reveals a bending of the $\alpha \beta$-tubulin heterodimer to ca. 12$^\circ$ (“The Determinants That Govern Microtubule Assembly from the Atomic Structure of GTP-Tubulin” 2011; Ayaz et al. 2012; Pecqueur et al. 2012). In our simulations the heterodimer at the plus end of a microtubule is very bent with respect to the next heterodimer in the PF. The bending angle is 13±5$^\circ$, which agrees with the aforementioned 12$^\circ$  observed experimentally as well as the average value of 11$^\circ$  for the simulated PF-sheet. Similarities in numeric values of bending angles support the hypothesis that the bending of the $\alpha \beta$-tubulin is an intrinsic property of the heterodimer.

It is also interesting to note that the tubulin bending at the minus end of the simulated microtubule is considerably smaller. The corner heterodimer at the minus end is bent 4±2$^\circ$ with respect to the neighboring heterodimer in the PF. It seems that the steric constraints, which arise upon binding to the neighboring heterodimer, are acting differently at plus and minus ends. To depart from straight orientation, the polymerized $\alpha \beta$-tubulin heterodimer should slightly rotate, which is observed by the PC-mode analysis (Fig.  7) and by visual inspection of the microtubule lattice (Figs. S5–S6 in Supplementary Information). In turn, the minus end corner heterodimer rotates in the same direction as the plus end heterodimer, thus forcing its neighboring heterodimers to twist the microtubule lattice and tend to cap the minus end. Apparently, a skewed interface between the $\alpha$- and $\beta$-monomers contributes significantly to the flexibility of the heterodimers within the lattice. This allows us to speculate that the role of GTP-binding pocket during polymerization is in controlling the axial rotation of $\alpha \beta$-tubulin heterodimers in the bound state. Both rotation and bending motions are needed to form the lattice of the microtubule. It is assumed that GDP-bound heterodimers are forming weaker lateral and longituidinal bonds compared to the GTP-bound ones (McIntosh et al. 2018). However, control of the axial rotation mode might explain differences upon binding to small molecules such as GDP, GTP and guanosine-5’-[($\alpha ,\beta$)-methyleno]triphosphate. Unfortunately, the data we present does not give overwhelming evidence of such an effect. Therefore, further investigations are required to elucidate this. Yet, we believe this observation is worth mentioning because it can provide additional insights into the mechanism behind the dynamic instability of the microtubule.

The tendency to cap the microtubule minus end we observed in our simulations may be an important reason for the high stability of the minus end. Capping reduces the possibility to attach another heterodimer next to the corner minus heterodimer due to lattice deformation. Capping ends are often encountered in microtubules when they are template-nucleated from $\gamma$-tubulin ring complex (Höög et al. 2011). A capped minus end in the absence of a template can potentially provide conservation of the minus end structure within minutes. Additionally, the capping tendency should be very strong because lattice deformation was revealed in a relatively short simulation compared to the microtubule growth time.

One can wonder if such a behavior of the microtubule can be affected by the presence of the high amount of Mg$^{2+}$ and Cl$^{-}$ ions. Figures S8 and S9 in Supplementary Information show the mass density distributions in *X*-, *Y*- and *Z*-directions for GDP- and GTP-bound microtubules, respectively. Mg$^{2+}$ ions are evenly distributed in the simulation boxes for both microtubules. On the profiles for Cl$^{-}$ ions a global minimum can be observed in the center of the GDP-bound microtubule for the profile in the *Z*-direction, while in the case of the GTP-bound microtubule the Cl$^{-}$ ions are more situated in the center of the microtubule rather than at its edges. Such a difference in the distribution of ions, which results in the nonidentical charge distribution in its turn, can be a reason for the divers dynamic behaviors of GDP- and GTP-bound microtubules: the GDP-binded microtubules shows faster relaxation than the GTP-binded one on the time-scale above 1 ns, which is confirmed by self-intermediate scattering functions.

We hope that our observations will contribute to further developments of the mechanistic models of the entire microtubule. Such results of atomistic simulations are proven to be valuable for the implementation of coarse-grained models of the microtubule (Theisen et al. 2012; Dima and Joshi 2008). Our finding on partial weight of the PC-modes in bound bio-assemblies is a clear example showing that results from single-molecule simulations should be corrected when the same molecule in a bound state is considered. Despite the diversity of microtubule models (Deriu et al. 2010; Zakharov et al. 2015; Chen and Hill 1983, 1985; Hill 1984; Bayley et al. 1990; Martin et al. 1993; Flyvbjerg et al. 1996; VanBuren et al. 2005; Stukalin and Kolomeisky 2006; Margolin et al. 2006; Brun et al. 2009; Ranjith et al. 2009; Padinhateeri et al. 2012), most of them totally discard the internal structure of the $\alpha \beta$-tubulin heterodimer. Yet, a truly comprehensive model should not only rely on an analytical description in matching certain experimental data, but it should also provide a full scale overview of the self-assembly, given its central role in cell biology (Thomas and Schwartz 2017).

## Conclusions

Growth and dynamics of microtubules have been a major focus of research efforts for decades. Despite the central role of the GTP-hydrolysis, the structural details of the self-assembly mechanism are still unknown. Most probably, the self-assembly is governed by structures of individual units, which makes structural studies of tubulin even more attractive for pharmaceutical applications, since they could give a good explanation regarding how various diseases are developed.

In the current work we investigated biologically relevant large scale motions, or essential dynamics, of $\alpha \beta$-tubulin heterodimers. Simulations were performed for two assemblies of tubulin, namely, a PF-sheet, which at a certain approximation represents the structure of a microtubule tip, and a complete short microtubule. Our modeling complements previous computational works done with microtubules (Wells and Aksimentiev 2010; Deriu et al. 2010; Zakharov et al. 2015; Chen and Hill 1983, 1985; Hill 1984; Bayley et al. 1990; Martin et al. 1993; Flyvbjerg et al. 1996; VanBuren et al. 2005; Stukalin and Kolomeisky 2006; Margolin et al. 2006; Brun et al. 2009; Ranjith et al. 2009; Padinhateeri et al. 2012) and closes the gap in knowledge obtained from MD simulations of an individual unpolymerized $\alpha \beta$-tubulin heterodimer and highly coarse-grained network models.

The observed motions are qualitatively similar to those obtained in simulations of an individual $\alpha \beta$-tubulin heterodimer, yet large discrepancies in the relative weights of the modes occur. The identified redistribution of the PC-modes from one system to another indicates that tubulin assembly/disassembly is in close relation to tubulin modes existing in a single heterodimer, or more generally to the structure encoded in the $\alpha \beta$-tubulin.

We detected only small bending of the minus end heterodimers. Additionally, the lattice of the microtubule tends to cap the minus end by twisting the corner heterodimer and its neighbors, which could potentially contribute to the higher stability of the minus end. The bending in the PF-sheet was large in the minus end, which suggests a noticeably different behavior for small tubulin constructs. The alteration in the motions of the minus end upon the growth of the microtubule nuclei might be the core reason for a switching between nucleation and fast growth phases in microtubule polymerization.

Distributions and variations of the large scale motions observed in the simulations give us motivation to revise the role of GTP and similar small molecules for the microtubule dynamics. Therefore, we propose that molecules bound to GTP-binding pockets are primarily affecting the rotational mode of the $\alpha \beta$-tubulin, although this suggestion requires more specific and extended simulations of polymerized microtubular fragments for confirmation.

Finalizing this work, we can say that the present atomistic MD simulations on a microsecond time-scale revealed mechanisms of the dynamics of microtubules.

As a future work, simulating microtubules of other lengths and with small molecules, proteins and enzymes, and modeling dysfunctioning microtubules would help to understand the behavior of these species when diseases occur and, perhaps, this could lead to a development of improved drugs.

## Supplementary Information

Below is the link to the electronic supplementary material.

Supplementary file1 (PDF 1788 KB)
