# Supplementary material for: Atomistic molecular dynamics simulations of tubulin heterodimers explain the motion of a microtubule
Source: Eur Biophys J. 2021 Jul 2;50(7):927–40. doi: 10.1007/s00249-021-01553-1 (PMC8448678; doi:10.1007/s00249-021-01553-1)
Supplement: Supplementary file 1 — Supplementary file1 (PDF 1788 KB) [file 249_2021_1553_MOESM1_ESM.pdf]

Atomistic molecular dynamics simulations of tubulin heterodimers explain the motion of a microtubule

Supplementary Information

Alexandr Nasedkin[a], Inna Ermilova*[a], Jan Swenson[a]

[a]Department of Physics,
Chalmers University of Technology, SE 412 96, Gothenburg, Sweden

E-mail to corresponding author: inna.ermilova@chalmers.se; ina.ermilova@gmail.com

Content

1. **Figure S1: values computed for dimers in GDP- and GTP-bound microtubules on different time intervals during the equilibration runs.**

2. **Figure S2: The matrix of inner product of eigenvectors for GDP- and GTP-bound microtubules.**

3. **Figure S3: Dependence of the inner product of the first eigenvectors on the number of concatenated trajectories used for covariance analysis.**

4. **Figure S4: Histogram of bending angles between heterodimers observed in the MD simulations of PF-sheet .**

5. **Figure S5: Microtubule lattice from the last frame of 50 ns simulations. The balls present centres of masses of individual monomers.**

6. **Figure S6: Microtubule lattice from the last frame of 50 ns simulations. The balls present centres of masses of individual monomers.**

7. **Figure S7: Self-intermediate scattering functions (SISFs), computed for backbones and side chains of proteins of GDP- and GTP-binded microtubules.**

8. **Figures S8-S9: Mass density profiles for ions in the simulations with GDP- and GTP-bound microtubule respectively.**

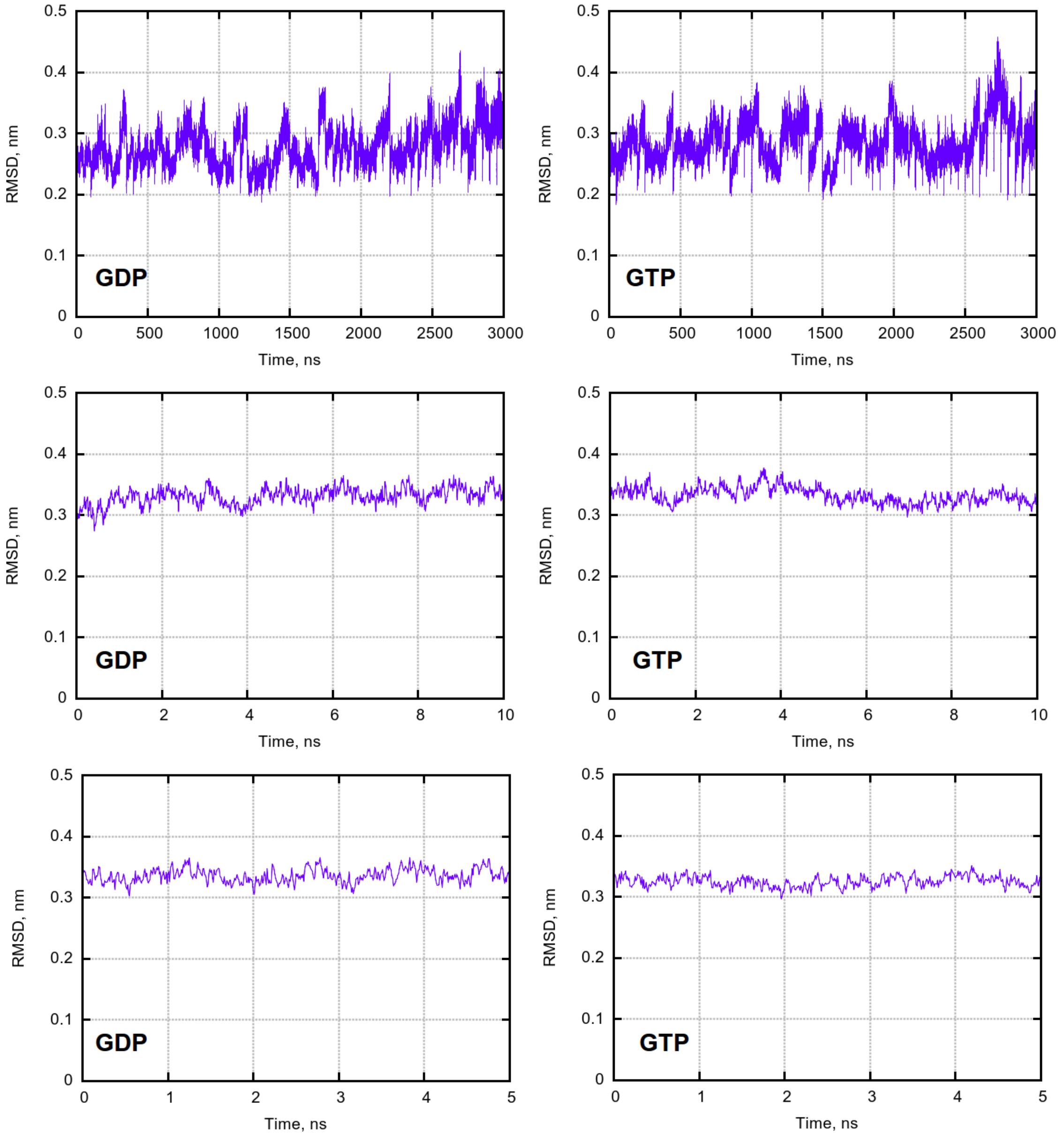

Figure S1: RMSD values computed for C_α atoms of dimers in GDP- and GTP-bound microtubules on different time intervals during the equilibration runs: last 3 μs, last 10 ns and last 5 ns.

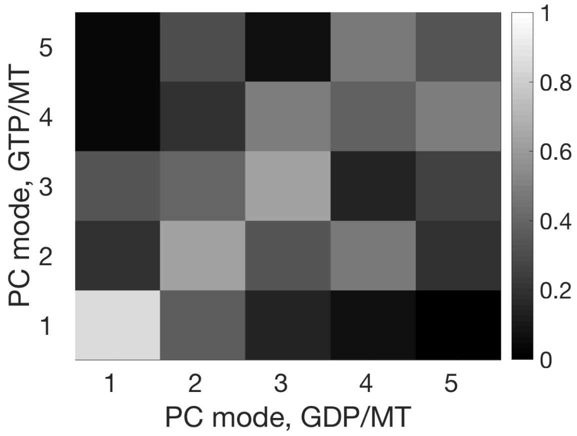

Figure S2: The matrix of inner product of eigenvectors for GDP- and GTP-bound microtubules. The first five eigenvectors are shown for every system. The higher value for the inner product indicate higher similarity between eigenvectors.

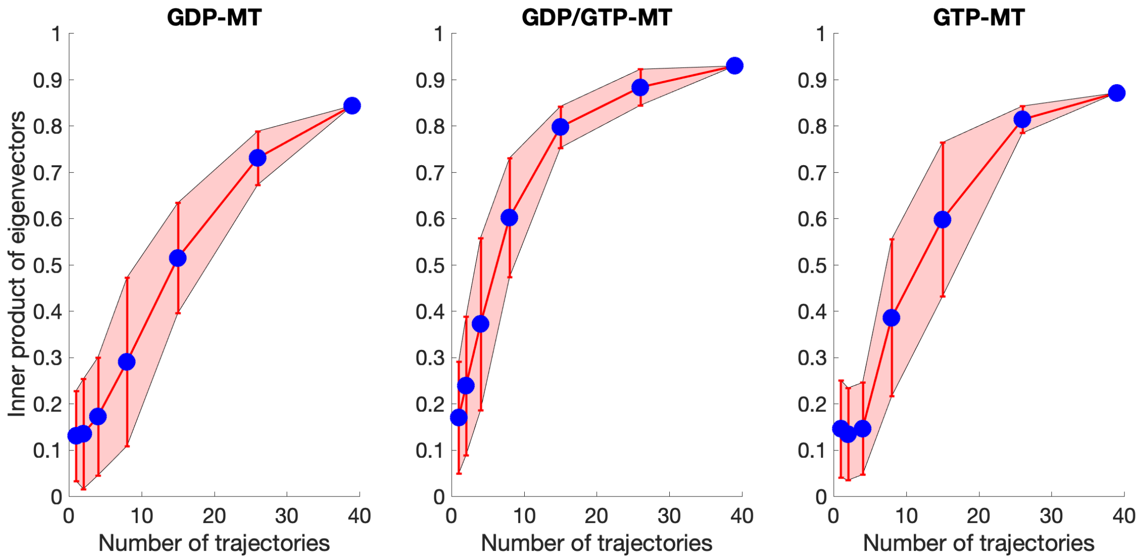

Figure S3: Dependence of the inner product of the first eigenvectors on the number of concatenated trajectories used for covariance analysis. The inner product of two eigenvectors is 1 for the identical eigenvectors and 0 for the orthogonal ones. The number of concatenated trajectories used: 1, 2, 4, 8, 15, 26 and 39. All possible combinations of concatenated trajectories of specific length were used to determine the inner product. Depicted error (red bars) is the standard deviation of all the inner products. Pink area limited to the error bars is the guide for the eye. Note that for N=39, there were only two trajectories analysed, resulting in one inner product. Thus, the error bar for N=39 is abscent.

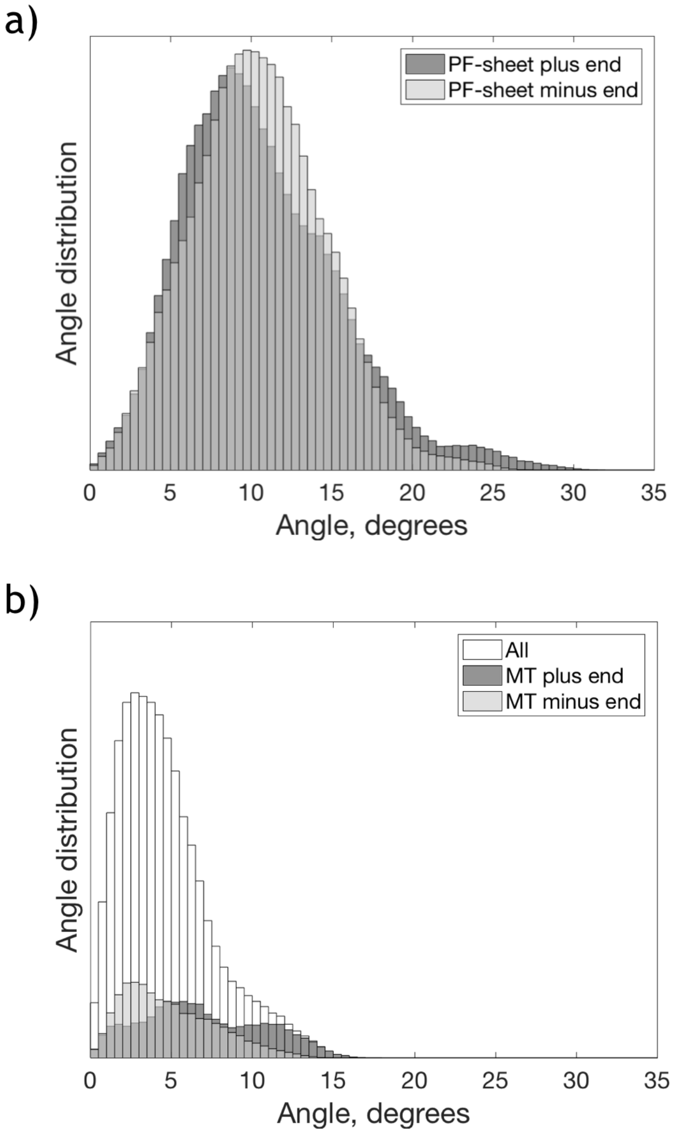

Figure S4: Histogram of bending angles between heterodimers observed in the MD simulations of PF-sheet (a), and microtubule (b). Areas in the histograms for plus- and minus ends of PF-sheet are equal. Areas in the histograms for plus- and minus ends of microtubules are each equivalent to 1/5 of the total distribution (All) because there are in total five full turns of angles between six turns of heterodimers.

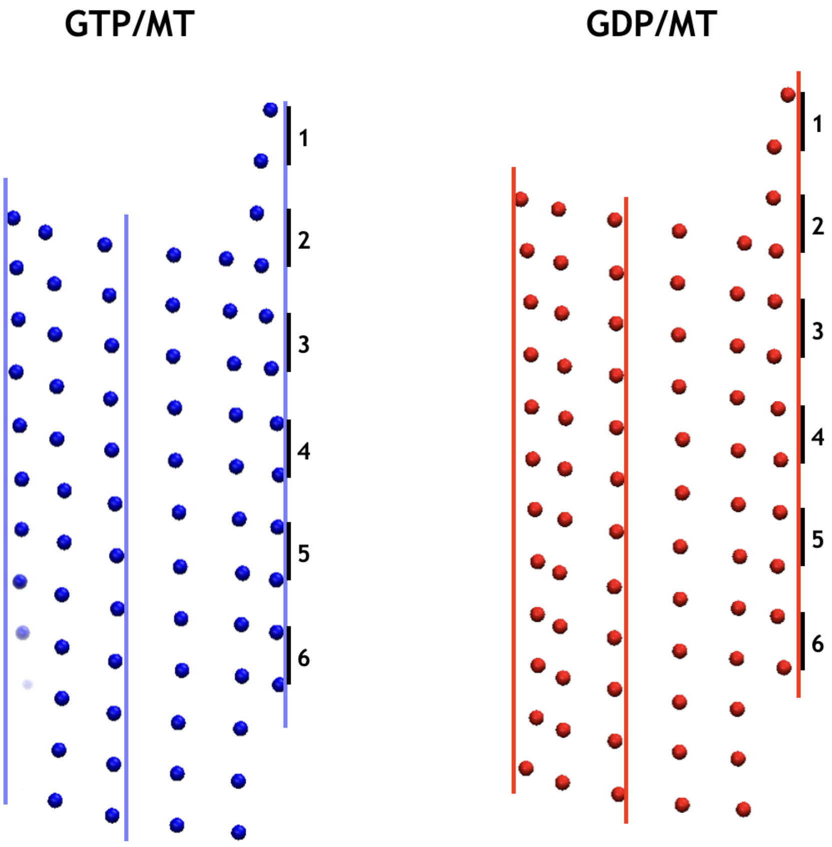

Figure S5: Microtubule lattice from the last frame of 50 ns simulations. The balls present centres of masses of individual monomers. Left panel is GTP-bound microtubule, right panel is GDP-bound. Straight lines is the guide for the eye. Numbers indicate individual heterodimers.

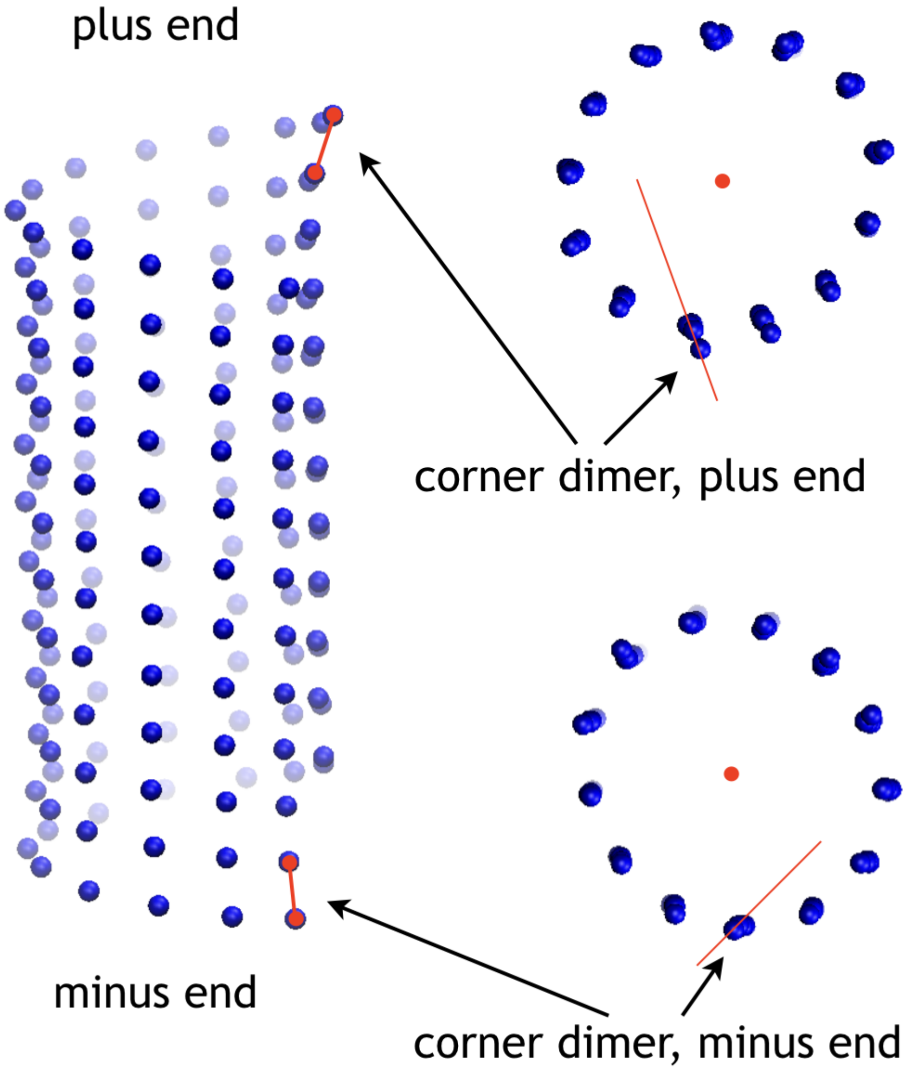

Figure S6: Microtubule lattice from the last frame of 50 ns simulations. The balls present centres of masses of individual monomers. Left panel is the side view, right panel is the view from the plus and minus ends. The red line connects centres of masses of monomers in the corner heterodimer. The purpose of this line is to show circumferential displacement of the corner heterodimer.

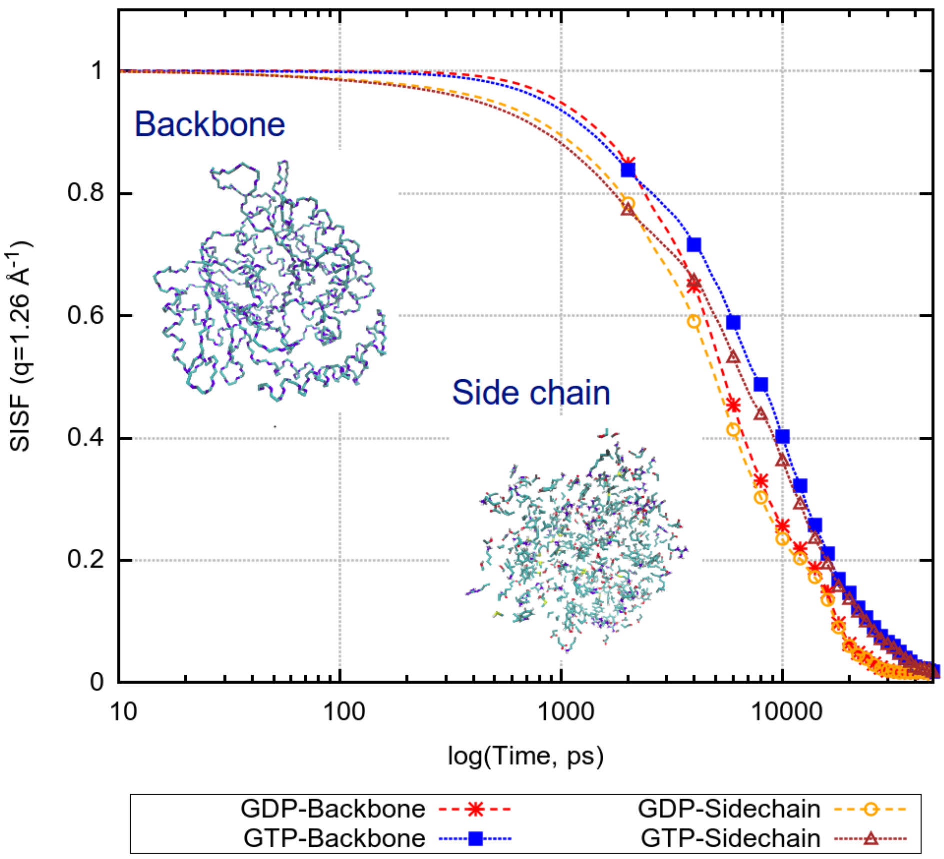

Figure S7: Self-intermediate scattering functions (SISFs), computed for backbones and side chains of proteins of GDP- and GTP-binded microtubules.

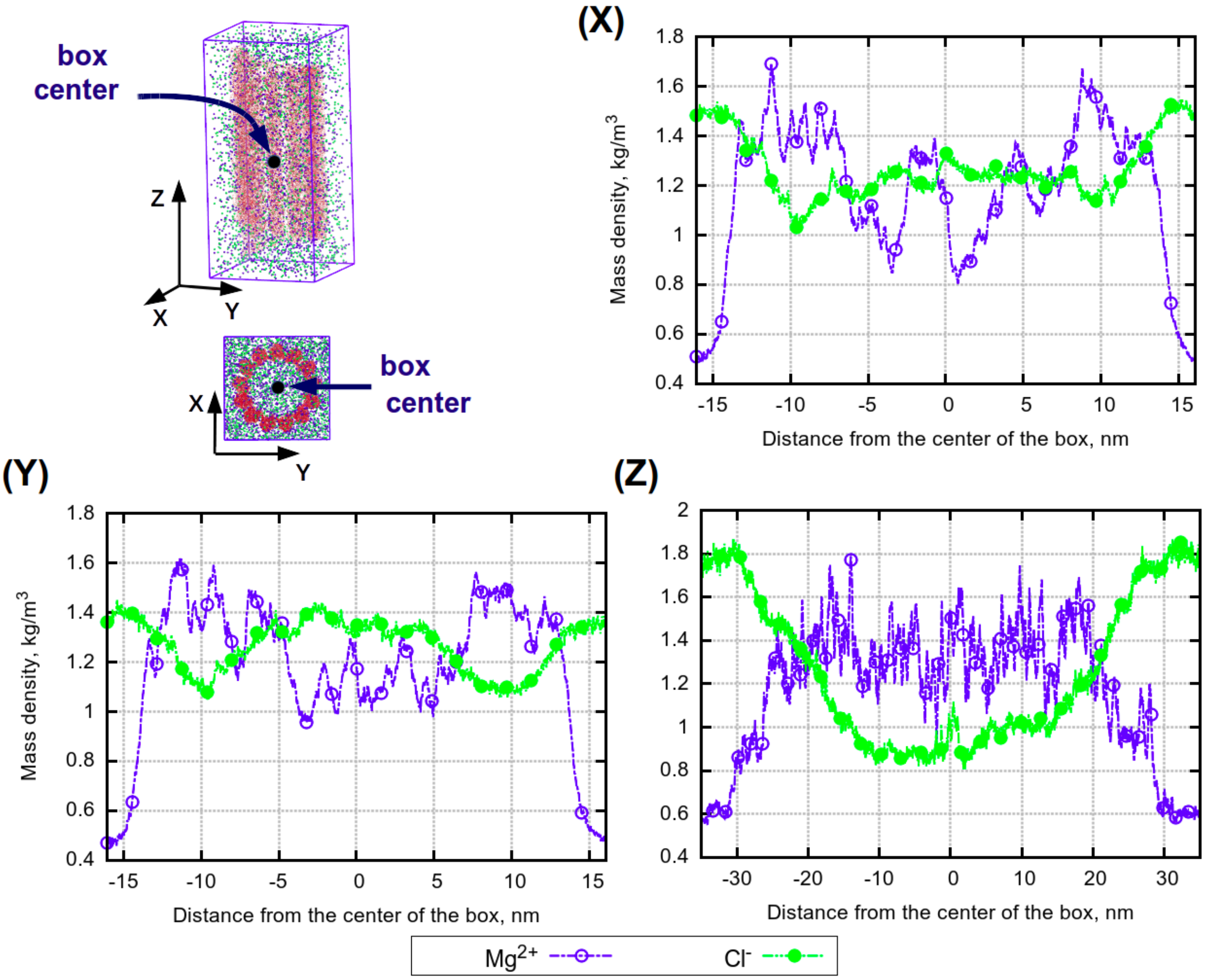

Figure S8: Mass density profiles for ions in the simulation with GDP-bound microtubule. X, Y and Z are directions in which those profiles were computed. Mg^{2+} ions are visualized as blue spheres, Cl^{-} ions are green spheres, the microtubule is visualized as red ribbons.

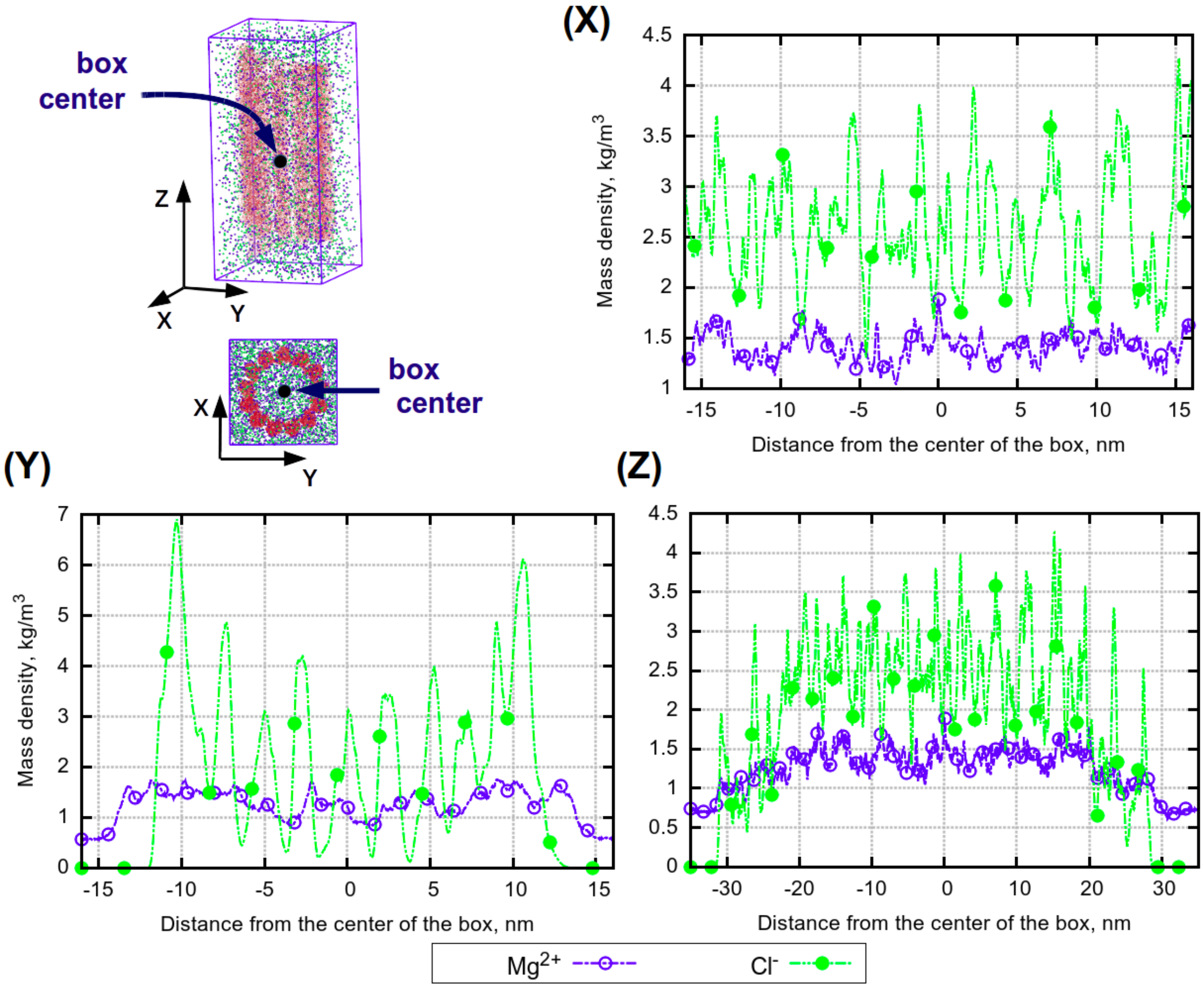

Figure S9: Mass density profiles for ions in the simulation with GTP-bound microtubule. X, Y and Z are directions in which those profiles were computed. Mg^{2+} ions are visualized as blue spheres, Cl^{-} ions are green spheres, the microtubule is visualized as red ribbons.
